# Opportunities and Threats of the Legally Facilitated Performance-Based Managed Entry Agreements in Slovakia: The Early-Adoption Perspective

**DOI:** 10.3390/healthcare11081179

**Published:** 2023-04-19

**Authors:** Petra Hospodková, Klára Gilíková, Miroslav Barták, Elena Marušáková, Aleš Tichopád

**Affiliations:** 1 Department of Biomedical Technology, Faculty of Biomedical Engineering, Czech Technical University in Prague, 272 01 Kladno, Czech Republic; hospopet@fbmi.cvut.cz (P.H.); miroslav.bartak@fbmi.cvut.cz (M.B.); ales.tichopad@fbmi.cvut.cz (A.T.); 2 Department of Public Health, Faculty of Health and Social Work, Trnava University, 917 01 Trnava, Slovakia; elena.marusakova@aopp.sk

**Keywords:** performance-based manage entry agreements, risk-sharing agreements, orphan drugs

## Abstract

Slovakia has adopted an amendment to Act No. 363/2011, regulating, among other things, drug reimbursement and is undergoing a significant change in the availability of innovative treatments for patients. High expectations are associated with arrangements related to performance-based managed entry agreements. Opinions and positions towards this change appear to be inconsistent, and for the further application of the law in practice and when setting up the main implementation processes, it is necessary to understand the positions and opinions of the individual actors who are involved in the PB-MEA process. The interviews were conducted in the period from 20 May to 15 August 2022 around the same time as the finalisation of the amendment to Act No. 363/2011 and its adoption. A roughly one-hour open interview was conducted on a sample of 12 stakeholders in the following groups: representatives of the Ministry of Health, health-care providers, pharmaceutical companies and others, including a health insurance company. The main objective was to qualitatively describe the perception of this topic by key stakeholders in Slovakia. The responses were analysed using MAXQDATA 2022 software to obtain codes associated with key expressions. We identified three main strong top categories of expressions that strongly dominated the pro-management interviews with stakeholders: legislation, opportunities and threats. Ambiguity and insufficient coverage of the new law, improved availability of medicinal products and threats associated with data, IT systems and potentially unfavourable new reimbursement schemes were identified as key topics of each of the said top categories, respectively. Among individual sets of respondents, there is frequent consensus on both opportunities and threats in the area of implementing process changes in PB-MEA. For the successful implementation of the law in practice, some basic threats need to be removed, among which in particular is insufficient data infrastructure.

## 1. Introduction

The reimbursement system in Slovakia has long been known as a restrictive one based on internal and external referencing, explicit willingness to pay anchored in the law and mandatory budget ceilings. Medicines that entered the market with significant price discounts, or their manufacturers or the holder of the registration authorization (both referred to as pharma), were refrained from entering the market. The availability of innovative medicines in Slovakia is significantly lagging behind other European countries [1,2].

In August 2022, Slovakia adopted an amendment to Act No. 363/2011 regulating, among other things, drug reimbursement. This is expected to be a significant change in the availability of innovative treatments for patients. The primary changes brought about by the amendment of the law are the change in the reimbursement of medicines, the cancellation of the conditional inclusion of drugs in the list of approved (referred to as categorized) medications and, last but not least, the transfer of responsibility for closing medication reimbursement contracts from health insurance companies to the Ministry of Health (MH) [3]. The reimbursement of drugs is measured by their clinical benefit; for holders of registration of innovative medicines, this means submitting a pharmaco-economic analysis to assess the medical need of the drug, financial efficiency and impact on the budget. Within the implementation of these legislative changes, there are high expectations on the part of stakeholders. In general, managed entry agreements can be described as a tool that gives patients access to new medicines while the risk of uncertain efficacy is being shared between health authorities and pharma [4,5,6,7]. This type of agreement considers uncertainty about the performance of technologies, particularly the performance of drugs, and regulates their adoption to maximize the impact of their use or to reduce the impact on the budget. The number of MEAs increases over time, especially in response to the high prices of new innovative drugs such as drugs for cancer and rare diseases, those often exhibit variable individual outcomes [8]. Performance-Based Managed Entry Agreements (PB MEAs) are a sub-type of MEAs in which the individual patient’s treatment success rate is monitored, and that rate determines the reimbursement amount. They present an alternative to financial MEAs. PB MEAs are certainly more innovation-friendly than financial MEAs, as the reimbursement for the respective drug, as set out in the agreement, is subject to the performance of the drug in its real-world use in patients as soon as the market entry is granted upon the specified PB MEA agreement. The way PB MEA works is that reimbursement is reduced or stopped altogether if the response to treatments is not successful. Information about the health outcome indicators that assess the product performance is mostly highly confidential, as well as most of the content of performance-based MEAs. Due to challenges such as implementing new systems as opposed to using existing data structures, experience with PB-MEA is essentially still limited [1,9,10,11,12,13,14]. The main concern for the widespread use of PB-MEA comes from a shortage of established data on parameters that indicate the performance of products, or from the lack of interpretation leading to little minimization of uncertainties in product effectiveness [15]. Other situations that may pose challenges for PB MEAs include when the treatment turns out to be less effective than originally anticipated, raising the question of terminating its coverage, how to deal with the increased administrative load of implementing PB MEA and the fact that the cost will increase for those involved in these arrangements [4,16,17]. Even though all MEAs are common, the most typical ones are financial agreements. PB MEAs are used only occasionally, mostly in the UK, Sweden and the Netherlands [1].

The exemption-based access used in Slovakia for medicines for seriously ill patients should be increasingly replaced by a permanent MEA- regulatory policy. The door for PB-MEA was opened by the amendment of Act No. 363/2011, which sets the conditions for the reimbursement of medicines. However, given the scale of the changes and current practice, there appear to be high expectations as well as concerns, particularly as performance measurement may be seen as an additional burden on top of the already mandatory budget ceilings.

Even though the new legislation is positively expected as a means to improve the availability of medicine on the market, concerns can be expected. The aim of our effort is to clarify and record the expectations and concerns due to the time when the amendment was adopted to guide future policy steps as well as reflect on expectations met and not met with regard to PB MEAs.

The remainder of this article is organized as follows. Section 2 explains the methodological approach, including the creation of a scenario, the selection of interviewees, and the method of data coding. Section 3 defines the number of interviewees and their field of work, presents the “top-level codes”, and the following three parts are devoted to the analysis of the issues (legislation, threats and opportunities) that seek to answer the research questions. The conclusion consists of a discussion of the results obtained, followed by the conclusions and limitations.

## 2. Materials and Methods

### 2.1. The Timing and the Scope

This was qualitative research undertaken to reveal the attitudes of the stakeholders with regard to PB MEAs in Slovakia. The interviews were conducted in the period from 20 May to 15 August 2022, i.e., at the time of the finalization of the amendment to the Act No. 363/2011.

We conducted a semi-structured qualitative interview with open questions (see Supplementary) among stakeholders with a professional or otherwise relevant relationship to the issue of availability, reimbursement and prices of medicines in Slovakia.

The selection of stakeholders and the interviews were initiated at a time when the final version of the amendment to Act No. 363/2011 was already known. It can be assumed that all interviewees were more or less familiar with its wording. The questioning did not focus in detail on the wording of individual provisions of this law, however, but rather on the concept of the PB MEA and its feasibility, risks and potential benefits for Slovakia.

### 2.2. Recruitment and Selection of Interviewees

We used purposeful and snowballing sampling to select stakeholders to participate in the study [18]. All stakeholders were approached through a standardized email invitation. The stakeholders were then sampled based on seniority and function, whereas senior representatives with a history of direct involvement in drug and medicinal product reimbursement were given preference in joining our study.

In the first phase, a map of potential stakeholders was generated and subsequently divided into four *occupational groups* as follows:

- Representatives of the MH (denoted as MH set),
- Health-care providers (HCP),
- Pharma business representatives, including one of their two local associations (Pharma),
- Others such as representatives of a patient organizations, HTA bodies or agencies and health insurance companies (Other).

### 2.3. The Interview

The key areas of the interview script focused on the following topics of interest defined prior to the interviews:

- Perceived legislation with regard to procedural, methodological and decision-making aspects of the amended act;
- Opportunities and threats related to PB MEA implementation;
- Future perspectives;
- Measurable outcomes definition.

Interviews were conducted by four interviewers, each using MS Teams. All interviews were recorded and transcribed verbatims, including non-literary and slang expressions, argot, etc. The interviews were anonymized, and all data representing specific names or titles leading to the informant’s identification were deleted. Individual interviews have been transcribed into text form.

### 2.4. The Codes and the Coding Tree

Open coding technique was applied with the aim to generate meaningful in vivo codes. The meanings in the interviews were analysed using the MAXQDA 2022 Analytics Pro 2022 software (version 22.2.1, VERBI SoftwareGmbH, Berlin, Germany) and meaning codes were then created on the basis of the statements and topics repeated and emphasised by the interviewee. Briefly, text as a sequence is broken into parts, herein referred to as text segments, and these are given names—the so-called in vivo codes. The first level in vivo codes were further coded at the second level. Both types of codes then defined individual categories and sub-categories.

Repeated listening and reading of the interviews by two of the authors generated a first preliminary coding tree consisting of top-level categories (codes) and a sub-categories. The final version of the coding tree from this initial tree emerged subsequently by rationalizing, merging, creating new hierarchies and other adjustments to the codes of the preliminary coding tree. Each author coded half the interview summaries and reviewed the other author’s coding for the remaining summaries. Any discrepancies in codes generated were resolved by consensus.

## 3. Results and Discussion

### 3.1. Recruitment and Interviews

A total of 20 stakeholders were contacted, of which 12 agreed to participate. The average interview length was 45 min. Of the 12 respondents, 3 could always be assigned to each defined occupational group (MH, HCP, Pharma, Others) on the basis of their occupational background. In the interests of anonymity, no more specific details of occupation or position in the organisation are given.

### 3.2. The Top-Level Categories

**Figure 1:**
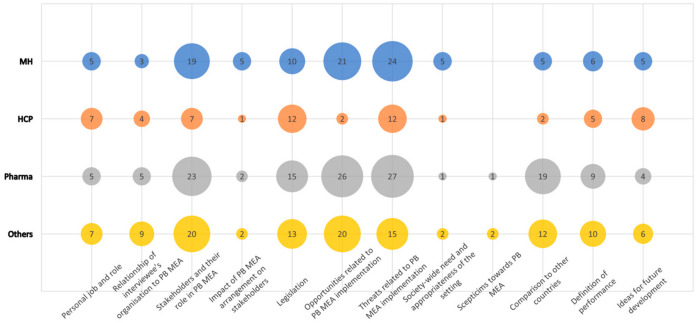
Frequency chart of codes in the top-level categories by occupational group.

The two-level coding hierarchy included 12 top-level categories comprising 112 sub-categories consisting of 424 text segments. The 12 empirically derived top-level categories together with the strength of their emphasis reported as the frequency of codes for each occupational group are shown in Figure 1. Of these 12 top-level categories, only 3 were within the scope of this paper and will be analysed deeper; the legislation, opportunities and threats related to the PB MEA implementation.

### 3.3. The Legislation

**Figure 2:**
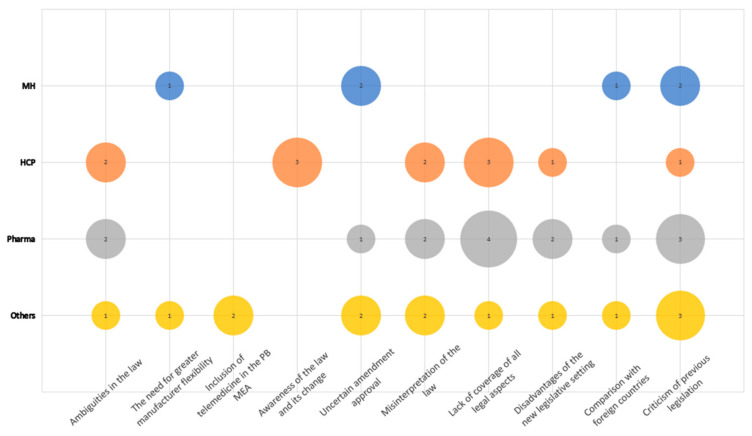
Legislation: frequency chart of codes in defined sub-categories within the category legislation by occupational group.

During the course of the interview, the interviewees from the Pharma frequently discussed the topic of the legislation. The strength of emphasis of the individual 10 sub-categories is illustrated in Figure 2. In total, 48 segments were identified.

Seven of the ten sub-categories on the topic of legislation have rather negative or pessimistic undertones. This can be illustrated by an HCP interviewee, stating: “I probably wouldn’t be able to work under these provisions and I’m not at all sure how the ideas of better accessibility …. are treated.” This was overwhelmingly prevalent among the interviewees from the Pharma, expressing their scepticism in four well-apparent sub-groups; two related to the insufficient low coverage and two rather generic sentiments expressing dissatisfaction such as, “The negative is that it will certainly affect other treatments that were otherwise available.” Complementary to the dissatisfaction of Pharma can be understood the reference to the need for greater manufacturer flexibility, which was made by representatives of MH and the Others group.

With very apparent reference to the current amendment to Act No. 363/2011, Coll., doubts were often expressed regarding the clarity of the law. This fact is evident from the two highlighted sub-categories: ambiguities in the law and misinterpretation of the law. Both sentiments were predominantly expressed by interviewees from the Pharma followed by HCPs and Others.

Insufficient coverage of all PB MEA aspects was also strongly emphasized by Pharma and reinforced by a specific reference to telemedicine software by other interviewees from the group of other stakeholders.

### 3.4. Opportunities Related to the PB MEA Implementation

**Figure 3:**
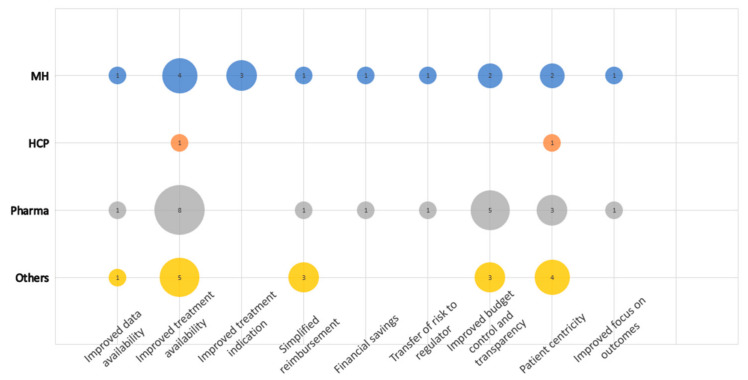
Opportunities: frequency chart of codes in defined sub-categories within the category opportunities related to the PB MEA implementation by occupational group.

There were nine sub-categories defined in the category Opportunities related to the PB MEA implementation, making it the second-largest category regarding the number of regulations (Figure 3). In total, 69 coded segments were identified.

Improved treatment availability and improved budget control and transparency were considered the main opportunities of the PB MEA mainly by Pharma, followed by Others. This can be well documented by the following statement: “The advantage is better availability of medicines for Slovak patients, while it will be possible to reach some agreement based on other categorization rules such as cost-effectiveness of the medicine.” The third dominant sub-category was Patient centricity. The first and the third sub-categories can be linked, as both are largely about the patient. Simplified reimbursement was further mentioned four times, mainly by Others.

### 3.5. Threats Related to the PB MEA Implementation

**Figure 4:**
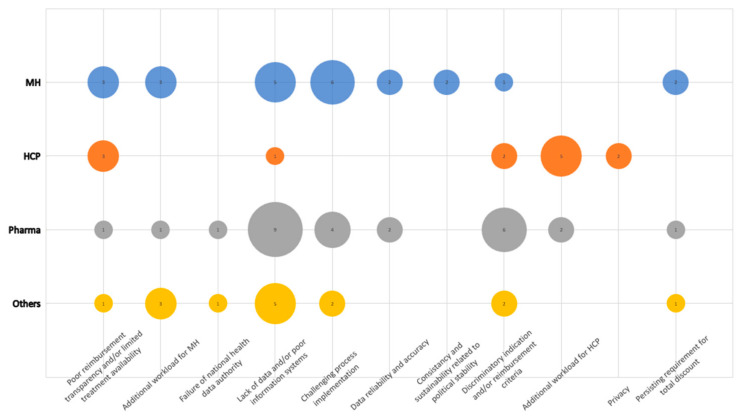
Threats: Frequency chart of codes in the sub-categories within the category threats related to the PB MEA implementation by occupational group.

In total, 11 sub-categories have been defined under this category. A total of 79 segments were identified (Figure 4). The dominant concern, as expressed by interviewees, was the inadequate availability of data and information systems to facilitate the PB MEA process, as well as a generally challenging implementation of the process itself. “Insurance companies often do not want to provide the data, claiming that the law does not require them to do so. Therefore, I see this as a bigger issue that affects performance-based contracts,” an interviewee from Pharma explained. These two issues may be linked to some extent, as the PB MEA process is known to be dependent on real-world data from existing systems and databases, such as administrative claims data or patient registries.

Strong technical concerns about the implementation of PB MEA were then fully contained in the following three separate sub-categories: lack of data and/or poor information systems, failure of national health data authority, and privacy. The first two domains are evident in the lack of hospitalisation data: “We do not have information on medications that are administered during hospitalisation when the patient is on the ward… It is covered by the hospitalisation package money or lump sum.”

Impact on the reimbursement process was considered a threat by Pharma, expressed mainly within the sub-category discriminatory indication and/or reimbursement criteria and data reliability and accuracy. Both sub-categories refer to collected patient data that do not accurately and reliably reflect the full and true benefit, for example because the definition of the therapeutic benefit is too narrow. Poor reimbursement transparency and limited treatment availability is, like the two previous sub-categories, an expression of concern about the implementation of drug reimbursement under the PB MEA system. Here, however, the concern is primarily from the perspective of physicians and MH. Given this concern was predominantly expressed in HCPs and the MH, it obviously rather applies to a potential discrimination from treatment in some patients who may show insufficient response to treatment in the decisive parameter but still benefit in another way.

The sub-category persisting requirement for total discount is the specificity of this amendment to the law, which, despite the introduced element of measuring the therapeutic benefit, still assumes that a discount is provided on the medicine without any reflection of the said benefit. Pharma will always be asked to provide a total discount when entering any MEA. These two elements in the latest amendment of the law are seen as contradictory. In addition, an interviewee from a health insurance company referred positively to the reinforced role of pharmacoeconomics as follows: “From the point of view of the health insurer, we see it as positive that criteria have been developed for innovative drugs and drugs for rare diseases, simply that the cost-effectiveness of each drug will have to be assessed… which has not been the case so far. For every single drug, including drugs that have a budget impact of less than 1.5 million, a pharmacoeconomics and budget impact analysis will be required. So, we see this very positively.”

The last obvious risk accompanying the implementation of PB MEA according to the latest amendment to the law is the expectation of more work for MH and HCPs.

Consistency and sustainability related to political stability is a stand-alone concern arising from the current perceived political reality resulting from instability in the government coalition.

## 4. Discussion

The health care system in Slovakia originally met the criteria of the Bismarck model [19]. In 2004, it was additionally inspired and changed according to the German healthcare system. A universal healthcare system is achieved through compulsory basic public and private insurance, which is regulated and substituted by the government through the Ministry of Health [20].

The Ministry of Health, through the process referred to as categorization, decides as well on the entry of medicines on the market and its reimbursement level [2]. This competence is now supplemented for selected highly innovative new medicines by their management of MEAs, including also PB MEAs. The amendment to the law introduced the obligation to conclude MEAs for all incoming innovative medicines [21] and for all medicines intended for the treatment of rare diseases [22]. Therefore, only a small proportion of new medicines are expected to be affected. From the point of view of expected costs, however, it will probably be about medicines with high entry prices and at the same time with great uncertainty from the point of view of expected future outcomes. In addition to the Ministry of Health, in Slovakia, health care payers, especially the state-owned general health insurance company, also plays a key role in ultimate medicine availability. The position of both these key players may differ though. The conservative view of payers may place a higher emphasis on the overall budget impact, while the Ministry can put more emphasis on the need to improve the availability of innovative medicines for Slovak patients. Thus, there is a certain distortion of attention, which is also perceived by the professional public. In addition to this rather economic dimension of the assessment of PB MEAs and their benefits and risks, there are also concerns about technical feasibility, as expressed throughout our research. A correct and fair evaluation of PB MEAs requires reliable data on the performance of the drug in patients, ideally at the individual level and for all patients in the country. The meanings conveyed in the interview mainly related to the area of potential controversy over the PB MEA between Pharma and the MH.

Our results show that Slovak stakeholders are expecting the amendment to the law with hope but are also aware of the threats. Most importantly, scepticism persists about the technical ability to support reliable data for PB MEA decision making at the individual product level. In particular, Pharma is concerned that the reimbursement process itself will become unclear, or that reimbursement will narrow to patients with narrowly defined benefits. This is undoubtedly a legitimate concern, as one of the main benefits of PB MEA is that it creates an incentive to target treatment primarily at benefitting patients. This raises the question of whether PB MEAs would actually lead to a shrinking market for companies. We think that in the aggregate, any reduction in the number of eligible patients should be compensated by a greater openness of the country to new products and new indications. This would lead to better availability of the drug and the simultaneous administration of the drug to patients to achieve the best possible therapeutic effect. Communication to the doctors should then take place accordingly.

One of the problematic topics mentioned in our research is the subject of full discounts on medicines. This mechanism is basically without a favourable therapeutic impact and represents a financial bottleneck for companies to enter many products. It would therefore be worth considering combining it with the principle of reimbursement for performance. The total discount for a given product on the national market could thus have several levels, where the total discount after the end of the period under review would be adjusted on the basis of the level of performance achieved. The overall ceiling on the insurance company’s costs would never be exceeded. The achievement of the ceiling should be a motivational benchmark for the producer. Conversely, penalisation in the form of discounts would be implemented where a defined proportion of treated patients do not achieve therapeutic outcomes. Alternatively, there would be an integrated model for calculating the discount according to the proportion of responders. Of course, it would also be possible to work with some intermediate producer of discounts for partial responses. It is evident that however much effort has been made to prepare the new law in a way that will improve the market entry of other drugs, there are significant concerns on the Pharma side. However, Pharma’s concerns may be justified, as the amendment was made with an awareness of the need not only to improve market access, but also to maintain strict control over costs. In the sub-category legislation, the MH’s reference to the need for more flexibility in Pharma is evident. This flexibility can then be understood as the need for companies to take risks related to PB MEA. One may also wonder to what degree the new legislation can be understood as supporting the PB MEA process, if there is still an obligation to cap the budgets impacts of individual drugs and if mandated discounts are negotiated prior to any real-world performance data is available. The PB MEA could thus find a legitimate advantage for all parties if it is the performance that significantly determines the level of discount. In this spirit, the discount would only be considered in cases where it turns out that the agreed performance is not achieved.

Although there is a high level of digitisation of medical records in Slovakia, it does not have sufficient flexibility for PB MEA purposes. Administrative data obtained for the purpose of reporting patient care are also not sufficient for monitoring performance in PB MEA, as they are missing clinical data. Other healthcare systems in the EU that are gradually introducing PB MEA are also dealing with similar difficulties [23].

It is well known that patients are involved in the earlier stages of medical innovation, for example in the design of clinical trials. Patient outcomes are also increasingly important in assessing the benefits of these innovations. Greater patient and public involvement at later stages could promote the adoption and diffusion of innovations [24]. The amendment under discussion has made it possible to use patient insights for the first time. Although the categorization of medicines is governed by the Ministry of Health, a collective decision-making body, the Categorization Committee, prepares the documents and recommendations. As of August 2022, this body will now have a single patient representative with defined limits and rights. Therefore, PB MEAs are to some extent also judged by Slovak patients. It can be assumed that, with regard to PB MEAs, patients’ motivation will determine the results from a more humanistic point of view. Slovak patients do not seem to differ from patients who were asked about preferences for involvement in the development of health services [25]. For a long time, Slovakia was in desperate need for new innovative medicines for its patients. The need originated from a critical dearth of these medicines, which also caused Slovakia to fall far behind developed European countries such as the Czech Republic, which now serves as an inspiration. Despite all this, the amendment of the above-mentioned fundamental law on the drug reimbursement could potentially make Slovakia an actual example for neighbouring countries which are facing initial problems regarding the data infrastructure and the evaluation of PB MEAs. The amendment opens the possibility of Slovakia being one of the first countries in Central and Eastern Europe to successfully implement PB-MEA.

This analysis was carried out to nail down the expectations and risks associated with implementing PB MEA as perceived at the time of the preparation and implementation of threats about the amendment of Act No. 363/2011 Coll. Our plan is to follow up this analysis with a detailed stakeholder survey of the success of the implementation after the time necessary to crystallize the impact on the health system.

The limitations lie primarily in the small sample of interviewees available at a time when the new legislation had not yet been implemented and experts on reimbursement for health innovations were previously only aware of market access for financial agreements.

## 5. Conclusions

The main finding is that the interviewed stakeholders and main actors of the Slovak reimbursement system for medicinal products support the implementation of PB MEA and its introduction into general use. Nevertheless, there are a few preconditions and necessities for systemic changes for successful implementation of such a challenging management tool as PB MEAs. The systemic changes include better data availability and long-term political stability. The detected threats are not impossible to resolve, they are more concerns based upon the existing experience with the status quo. Therefore, without consequential changes in drug utilisation and reimbursement at all system levels, PB MEAs cannot work. There is more than one view on what the implementation of PB MEA entails and what needs to be done to make it change for the better. At the level of Pharma, the doctor and the patient, there is a need for the strict and rational use of medicines based on the indication and reimbursement criteria for overall performance. From the perspective of the regulator and the payer, process stability, transparency and data availability must be secured, and a cost control achieved with PB MEA. Future research is needed to evaluate the results of this study in middle and long-term run based on the law implementation experiences and its impact on improving the health of patients and improving the effectiveness of treatment. Probably the biggest challenge for PB MEA in Slovakia is the availability of reliable data. This is viewed with great scepticism. This is why it would be advisable to implement small projects first, which is also the view of the MoH representative, “Given the data that are available now, I cannot imagine that functional performance-based agreements will be concluded. Perhaps for a drug that is an ultra-orphan and will be for 10 to 20 patients. Then it can be monitored. But in the case of many patients, I can’t imagine that happening.”

## Data Availability

Data are contained within the article or Supplementary Materials.

## Supplementary Materials

The following supporting information can be downloaded at: https://www.mdpi.com/article/10.3390/healthcare11081179/s1, File S1. PB-MEA scenario.
