# Supplementary material for: Opportunities and Threats of the Legally Facilitated Performance-Based Managed Entry Agreements in Slovakia: The Early-Adoption Perspective
Source: Healthcare (Basel). 2023 Apr 19;11(8):1179. doi: 10.3390/healthcare11081179 (PMC10138524; doi:10.3390/healthcare11081179)
Supplement: Supplementary file 1 [file healthcare-11-01179-s001.zip › healthcare-2308645-supplementary.pdf]

PB-MEA scenario

1. Opening question

Do you agree/disagree with managing the recording?

What is the role of your organization that you represent to PB-MEA?

3. Perceived legislation setting

How do you view the current draft legislation regarding PB-MEA?

Where do you see its shortcomings?

Is there an objective approach to all stakeholders?

Does it reflect societal needs, and is it appropriately set up? How do you perceive the societal need?

Do you perceive the legislation's procedural, methodological, and decision-making aspects as sufficient? (If now/why?)

4. Opportunities and threats of implementing PB-MEA

Where do you see the main threats to individual contracts of the parties to the agreements?

Where do you see the main opportunities/development potential for the parties to the agreement?

5. Future perspectives

Where do you propose possible cultivation directions of current legislation and planned implementation?

6. Outputs definition

In what way and on what basis should the results be defined?

Given the current data available in Slovakia, how do you view the implementation of PB-MEA?
